# Epidemic Trends and Spatial Distribution Characteristics of Hepatitis B in China: Surveillance Study

**DOI:** 10.2196/70888

**Published:** 2025-05-12

**Authors:** Xiaoxue Li, Jie Liang, Lanqing Ma, Shijie Shen, Xueying Han, Tao Sun, Heng Guo

**Affiliations:** 1 Department of Public Health, Shihezi University School of Medicine, Xinjiang, No. 59, North 2nd Rd, Hong-Shan District, Shihezi, 832000, China, 86 18909932079; 2 Key Laboratory for Prevention and Control of Emerging Infectious Diseases and Public Health Security, The Xinjiang Production and Construction Corps, Shihezi, China; 3 Key Laboratory for Major Infectious Disease Monitoring in the Northern Frontier Region of the Corps, Shihezi, China

**Keywords:** hepatitis B, joinpoint regression analysis, APC model, spatial epidemiology, public health, vaccination

## Abstract

**Background:**

Hepatitis B is an important public health challenge facing China. Understanding the long-term epidemiological trends and evolving spatial distribution patterns is critical for optimizing prevention strategies and achieving the World Health Organization’s 2030 hepatitis elimination targets.

**Objective:**

This study aimed to explore the epidemic trends and spatial distribution characteristics of hepatitis B in China from 2004 to 2020.

**Methods:**

This study used data on hepatitis B incidence from 2004 to 2020 from the China Public Health Science Data Center to analyze the time trend of hepatitis B incidence by joinpoint regression. The age-period-cohort model was used to analyze the age, period, and cohort effects of hepatitis B onset. Spatial autocorrelation analysis was used to explore the spatial distribution characteristics of hepatitis B in China.

**Results:**

From 2004 to 2020, China reported a total of 17,449,842 cases of hepatitis B, with an average annual incidence rate of 76.30/100,000. The incidence of hepatitis B in China showed an increasing trend from 2004 to 2007, with an average annual percentage change (AAPC) of 9.49 (95% CI 2.12-17.39), and a decreasing trend from 2007 to 2014, with an AAPC of −3.77 (95% CI −5.93 to −1.55). The incidence of hepatitis B in China tended to be stable from 2014 to 2020, with an AAPC of −0.46 (95% CI −2.86 to 2.01). Age, period, and cohort effect significantly affect the incidence of hepatitis B. The age effect showed that the incidence rate of hepatitis B reached its peak at the age of 22 years, with an average incidence rate of 176.173/100,000. The period effect showed that the highest level during the study period occurred during 2004‐2006. The cohort effect showed that the risk of hepatitis B increased first and then decreased, with the turning point of 1924‐1974. The incidence of hepatitis B varies significantly among regions. The incidence in the northeast and northwest regions has decreased, that in the south and southwest regions has increased, and that in other regions has remained stable.

**Conclusions:**

China has achieved remarkable results in the prevention and control of hepatitis B, but there are still differences in the incidence rate among different age groups and regions. These results suggest the need to further strengthen the identification and screening of high-risk populations and promote supplementary adult hepatitis B vaccination. Future intervention strategies should fully consider regional differences, implement precise intervention measures based on the epidemic trends and spatial distribution characteristics of each region, optimize resource allocation, and enhance the overall effectiveness of hepatitis B prevention and control.

## Introduction

Hepatitis B is a chronic infectious disease caused by hepatitis B virus (HBV) infection. Its onset is hidden and it is difficult to cure. Many infected people have no obvious symptoms in the early stage, and delayed treatment may lead to serious complications [1]. Hepatitis B is still an important public health problem in the world. According to the World Health Organization, by 2022, a total of 254 million people worldwide were infected with chronic hepatitis B, with 1.2 million new infections and 1.1 million deaths; the main causes of death were cirrhosis and hepatocellular carcinoma (HCC) [2]. HCC is the sixth most common cancer and the third leading cause of cancer death worldwide [3]. HCC is also the fourth most common cancer and the third leading cause of cancer death in China [4]. In China, about 85% of patients with HCC have a history of HBV infection. HBV can induce HCC in various ways, and it is the most common and important factor in induced HCC [5]. HBV infection and its complications have brought a heavy economic burden to patients’ families and society [6]. Therefore, understanding the epidemic characteristics of hepatitis B is helpful to provide a key basis for the optimization and precise implementation of hepatitis B prevention and control strategies.

Since China introduced the hepatitis B vaccine into the neonatal immunization program in 1992, the spread of hepatitis B has been controlled to some extent. The positive rate of hepatitis B surface antigen (HBsAg) in children under 5 years old has decreased from 9.9% in 1992 to 0.3% in 2014 [7]. However, due to China’s large population base, HBV infection is still serious, and China’s achievement of the global goal of eliminating hepatitis by 2030 is still hampered [8,9]. It is estimated that 90 million people are enduring chronic HBV infection in China, accounting for about 7% of the national population, and 330,000 people die of HBV-related cancer every year [10]. Although China has made some progress in hepatitis B control, it is still the country with the largest burden of HBV infection in the world. A series of health problems caused by HBV still pose a serious challenge to public health, and it is still necessary to continue to pay attention to high-risk regions and high-risk groups [11]. Therefore, gaining a comprehensive understanding of the epidemic trends and spatial distribution characteristics of hepatitis B incidence in China holds utmost significance for devising scientific, precise, and effective intervention strategies. It is pivotal for further optimizing China’s hepatitis B prevention and control system, as well as driving deeper advancements in prevention and control efforts.

Current research on hepatitis B transmission, both domestically and internationally, predominantly concentrates on maternal-fetal prevention strategies and immunization programs. However, there remains a paucity of studies investigating the age-period-cohort effects on hepatitis B risk stratification. Furthermore, existing epidemiological investigations are largely confined to localized regions, specific demographic cohorts, or short-term observational periods, lacking systematic assessment of spatiotemporal dynamics and nationwide epidemiological patterns of hepatitis B in China. However, the spread of infectious diseases may vary greatly due to different spatial distributions, which makes the spatial distribution characteristics important factors to consider when formulating the response measures for infectious disease outbreaks [12]. For this reason, researchers use geographic information systems to examine the spatial distribution of various infectious diseases. Spatial autocorrelation analysis is a common method to analyze the spatial distribution characteristics of diseases and is widely used in the field of public health, especially the spatial cluster analysis of infectious diseases. This approach enables the identification of disease clusters, visualization of incidence and clustering trends over time and space, and clarification of geographical distribution patterns and risk levels across different regions [13].

In this study, the joinpoint regression model was used to analyze the long-term trend of the incidence of hepatitis B in Chinese residents from 2004 to 2020. The age-period-cohort model was used to estimate the effect of age, period, and birth cohort on the risk of hepatitis B. The spatial autocorrelation analysis was applied to visualize and spatially aggregate the incidence of hepatitis B in China from 2004 to 2020, and analyze the distribution characteristics of hepatitis B in different regions of China. This study aims to understand the spatiotemporal trends of hepatitis B in different regions of China and the impact of age, period, and birth cohort effect on the risk of hepatitis B, to provide a basis for clarifying the risk of hepatitis B in China and implementing accurate and effective prevention and control strategies. The results of this study will provide key evidence for the optimization of the hepatitis B prevention and control system in China, provide a scientific reference for the rational allocation of medical and health resources, and also contribute methodology and practical experience for the global realization of the World Health Organization’s 2030 goal.

## Methods

### Data Source

Hepatitis B–related data in this study were sourced from the public database of the Public Health Science Data Center, which is hosted by the Chinese Center for Disease Control and Prevention [14]. The database is China’s official authoritative public health data platform. Relevant data are collected based on China’s notifiable infectious diseases reporting system, which requires medical institutions at all levels to confirm and report hepatitis B cases by the diagnostic criteria and management principles stipulated by the National Health Commission of the People’s Republic of China. The dataset contains the hepatitis B data of 31 provinces, autonomous regions, and municipalities directly under the central government from 2004 to 2020 (excluding Hong Kong, Macao, and Taiwan). The data include the annual incidence and number of cases of hepatitis B in different regions and age groups in China, ensuring the comprehensiveness and representativeness of the study. The demographic data of China and its provinces in the same period (from 2004 to 2020) were obtained from the China Statistical Yearbook [15]. China’s provincial vector maps are sourced from the National Basic Geographic Information System of China.

### Joinpoint Regression Analysis

The Joinpoint regression software (version 5.0.2), developed by the National Cancer Institute, was used to analyze the temporal trend of hepatitis B incidence in China from 2004 to 2020. Joinpoint regression software uses the default grid search method to construct the model and uses the Monte Carlo permutation test method for model optimization. The Bayesian information criterion served as the criterion for evaluating the quality of model fit. Joinpoint regression analysis segments the study period into various intervals by identifying multiple connection points, fits and optimizes trends within each interval, and thereby assesses the characteristics of hepatitis B incidence changes across different intervals. The number, location, and *P* value of the connection points were determined through a Monte Carlo permutation test (α=.05, 2-sided test). The average annual percentage changes (AAPCs) of the incidence rate of hepatitis B from 2004 to 2020, along with 95% CIs, were calculated. When AAPC>0 (*P*<.05), it indicates an increasing trend in incidence; when AAPC<0 (*P*<.05), it suggests a decreasing trend; and when *P*≥.05, it suggests a stable trend. The AAPC calculation formula is as follows:


(1) $$n\left(y|x\right)=b_{0}+b_{1x}$$

(2) $$AAPC=\left\{exp\left(\frac{w_{i}b_{i}}{w_{i}}\right)-1\right\}\times 100\%$$

x represents the year, y denotes the incidence rate, b_0_ signifies the intercept, b_i_ represents the regression coefficient, which is the slope coefficient of each line segment, and w_i_ indicates the number of years encompassed in each time segment.

Based on China’s geographical characteristics and administrative divisions, this study divided China into 7 regions: the northern region (including Beijing, Tianjin, Hebei, Shanxi, and Inner Mongolia), the northeast region (including Heilongjiang, Jilin, and Liaoning), the northwest region (including Shaanxi, Gansu, Qinghai, Ningxia, and the Xinjiang), the eastern region (including Shanghai, Jiangsu, Zhejiang, Fujian, Shandong, Anhui, and Jiangxi), the central region (including Henan, Hubei, and Hunan), the southern region (including Guangdong, Hainan, and Guangxi), and the southwest region (including Chongqing, Sichuan, Guizhou, Yunnan, and the Xizang). It also analyzed the trend of hepatitis B incidence rate across these 7 regions.

### Age-Period-Cohort Model

The age-period-cohort-model (APC model) is widely used to analyze incidence trends in epidemiology. Among them, the age effect reflects the difference in the risk of disease in different age groups. The period effect reflects the overall change in the incidence risk of all age groups at a specific time point. The cohort effect reflects the change in the risk of morbidity in people with the same birth year. This study used the online network analysis tool developed by Rosenberg, Check, and others to construct the APC model [16].

Due to the scarcity of cases among individuals aged 84 years and older, as well as the challenges in determining birth cohorts, this study adopted a 5-year age interval, dividing the age range of 0‐84 years into 17 age groups. The data from 2004 to 2020 were divided into 5 groups: 2004 to 2008, 2009 to 2013, 2014 to 2018, and 2019 to 2020, to construct the APC model.

The model uses the Poisson log-linear model and uses the Akaike Information Criterion for a comprehensive evaluation of the model’s goodness of fit. The formula for this model is expressed as:


(3) $$log\left(E_{ij}\right)=log\left(P_{ij}\right)+\mu +\alpha_{i}+\beta_{i}+\gamma_{k}$$

In this formula, E_ij_ and P_ij_ represent the total number of individuals who developed the disease in the ith age group during the jth period group, respectively. μ denotes the intercept term of the incidence rate. α_i_ signifies the regression coefficient for the ith age group, while β_i_ indicates the regression coefficient for the jth period group. γ_k_ stands for the regression coefficient of the kth birth cohort group.

### Spatial Autocorrelation Analysis

In this study, ArcGIS 10.8 (Esri) software was used to describe the spatial distribution of hepatitis B in China from 2004 to 2020 through global spatial autocorrelation analysis. In addition, Moran’s *I*, a global spatial autocorrelation coefficient, was used to assess the spatial distribution pattern of hepatitis B across various regions in China. Moran’s *I* values range from −1 to 1. A positive value indicates spatial clustering, with a larger value indicating stronger spatial clustering. Negative values indicate spatial dispersion, with a smaller value indicating stronger spatial dispersion. A value of 0 indicates no spatial autocorrelation. The formula for calculating the Moran’s *I* is as follows:


(4) $$I=\frac{n\cdot \sum_{i=1}^{n}\sum_{j=1}^{n}w_{ij}\;\left(x_{i}-\overset{´}{x}\right)\left(x_{j}-\overset{´}{x}\right)}{\left(\sum_{i=1}^{n}\sum_{j=1}^{n}w_{ij}\right)\cdot \sum_{i=1}^{n}{\left(x_{i}-\overset{´}{x}\right)}^{2}}$$

In this formula, the incidence rate is represented by λ, the average incidence rate is represented by X, and the matrix of spatial weights is denoted as W_ij_, representing the proximity relationship between region i and region j.

Local Moran’s *I* was used to conduct a local analysis of the distribution of hepatitis B cases in each region, and the local autocorrelation type was determined by visualizing the local autocorrelation map. The aggregation types can be categorized into the following four types: “high-high,” “high-low,” “low-high,” and “low-low.”

### Ethical Considerations

The data of this study were obtained from the public database authorized by the public health science data center of the Chinese Center for Disease Control and Prevention. According to the relevant provisions of the international code of Biomedical Research Ethics, this study meets the exemption conditions for ethical review, mainly for the following 3 reasons: (1) the research data are completely anonymized public information and do not contain any personally identifiable information; (2) this study did not involve any form of intervention, nor did it collect or use human biological samples; and (3) the processing and publication of the research results will not have any impact on the clinical diagnosis and treatment plan of the original data provider.

## Results

### Overall Description of Hepatitis B in China From 2004 to 2020

**Figure 1.**
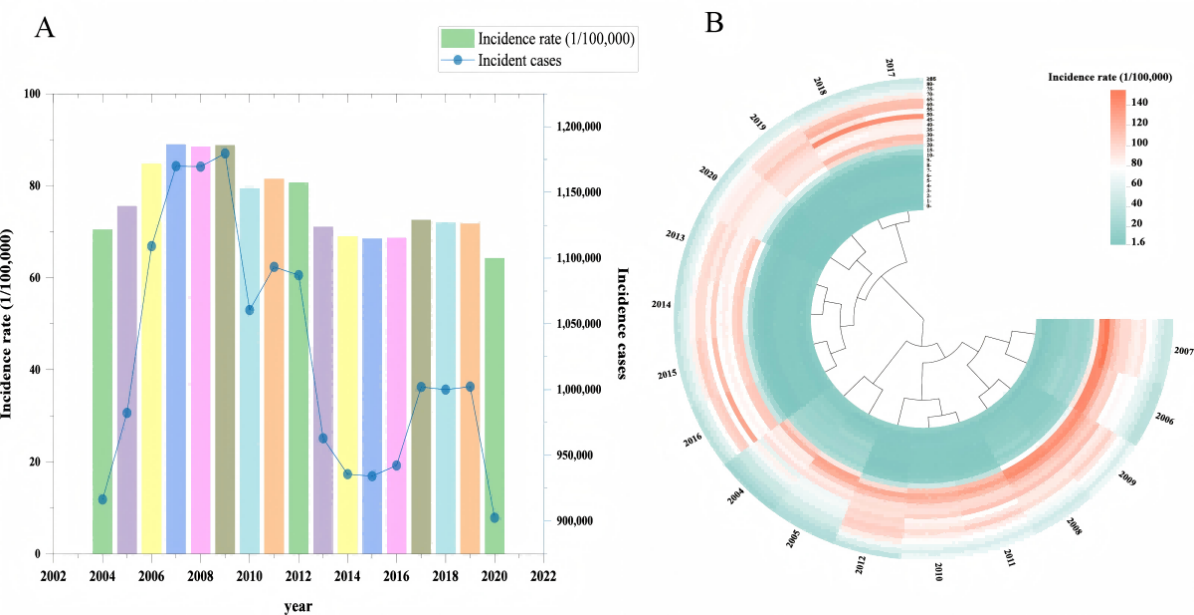
Incidence of hepatitis B in China from 2004 to 2020. (A) Several cases and incidence rate of hepatitis B in China from 2004 to 2020. (B) Heat map of age-specific incidence rates of hepatitis B in China from 2004 to 2020.

From 2004 to 2020, China reported a total of 17,449,842 cases of hepatitis B, with an average annual incidence rate of 76.3/100,000. The incidence rate fluctuated but exhibited an overall decreasing trend, decreasing from 70.5/100,000 in 2004 to 64.29/100,000 in 2020 (Figure 1A). From 2004 to 2020, the majority of hepatitis B cases occurred in the adult population, with 70.77% of cases falling within the age range of 20‐50 years (Figure 1B).

### Temporal Trends of Hepatitis B Incidence Rate in China From 2004 to 2020

**Figure 2.**
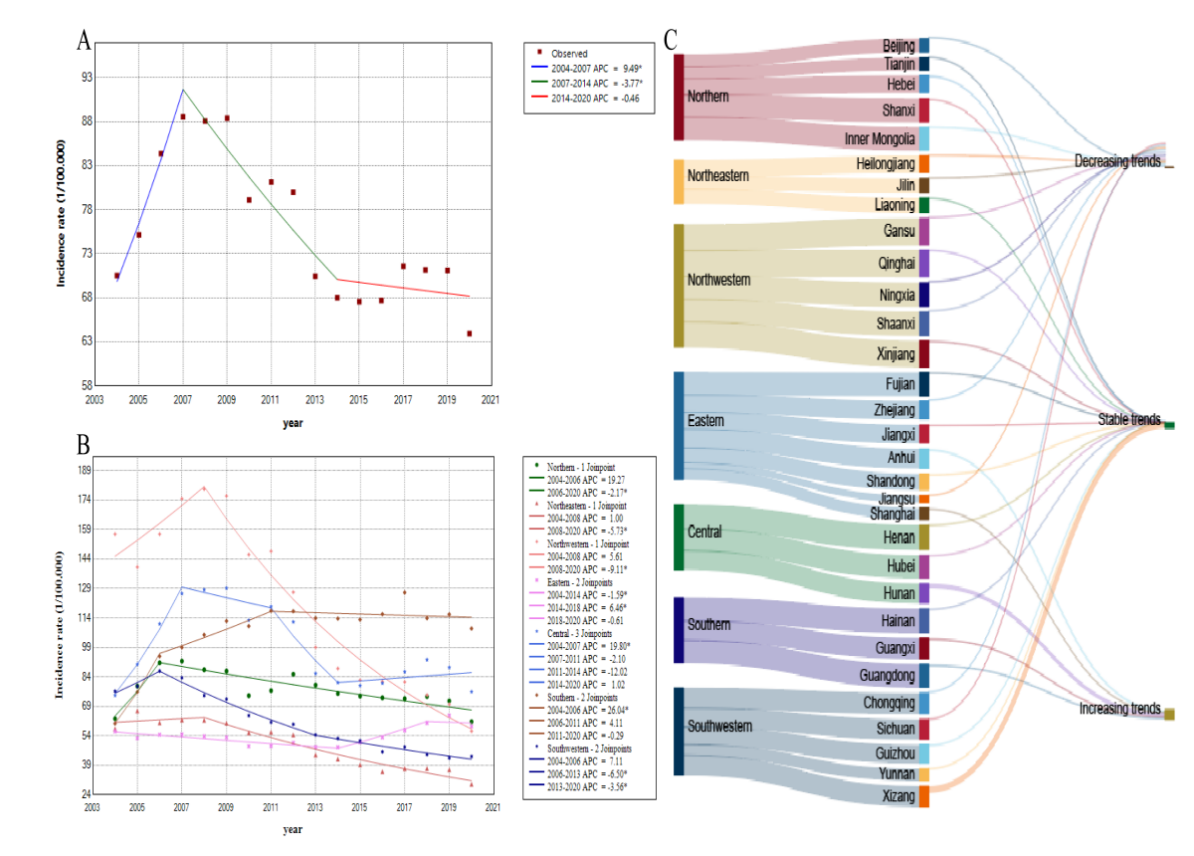
Temporal trends of hepatitis B incidence rate in China from 2004 to 2020: (A) temporal trends in the incidence rate of hepatitis B in China, (B) temporal trends in the incidence rate of hepatitis B across 7 regions in China, and (C) temporal trends in the incidence rate of hepatitis B across 31 regions in China. APC: annual percentage change.

The incidence rate of hepatitis B in China increased from 70.5/100,000 in 2004 to 89/100,000 in 2007 (AAPC=9.49, 95% CI 2.12 to 17.39). Subsequently, it decreased from 2007 to 69.05/100,000 in 2014 (AAPC=–3.77, 95% CI −5.93 to −1.55). From 2014 to 2020, the incidence rate of hepatitis B in China exhibited a stabilizing trend (AAPC=−0.46, 95% CI −2.86 to 2.01) (Figure 2A).

From 2004 to 2020, the incidence rate of hepatitis B decreased in Northeast China (AAPC=−4.09, 95% CI –5.60 to −2.56) and Northwest China (AAPC=−5.64, 95% CI −7.48 to −3.75). Conversely, the incidence rate of hepatitis B increased in Southern China (AAPC=4.07, 95% CI 1.41-6.80) and Southwest China (AAPC=3.60, 95% CI −4.86 to −2.33). However, the incidence of hepatitis B remained stable in the northern, eastern, and central regions (both *P*>.05; Figure 2B).

From 2004 to 2020, among all provinces, Xizang (AAPC=16.93, 95%CI 14.31-19.60) and Hunan (AAPC=10.52, 95% CI 7.91-13.20) experienced the most significant increases in hepatitis B incidence rates. Shaanxi (AAPC=−9.23, 95% CI −10.96 to −7.48), Chongqing (AAPC=−9.23, 95% CI −11.15 to −7.28), Beijing (AAPC=−9.05, 95% CI −15.36 to −2.26), Ningxia (AAPC=−9.84, 95% CI −11.37 to −8.28), Gansu (AAP=−8.31, 95% CI −12.63 to −3.77), and Zhejiang (AAPC=−8.43, 95% CI −11.25 to −5.52) saw a rapid decrease in hepatitis B incidence rates. The incidence rates of hepatitis B in other regions exhibited a stable trend (Figure 2C and Table 1).

**Table 1.** Joinpoint regression analysis of the incidence rate of hepatitis B in China from 2004 to 2020.

Region	AAPC^a^ (95% CI)		*t* test	*P* value
Northern region	0.29 (−1.85 to 2.47)		0.26	.794
Beijing	−9.05 (−15.36 to −2.26)		2.58	.009
Tianjin	−5.08 (−11.02 to 1.27)		−1.58	.114
Hebei	0.98 (−1.38 to 3.39)		0.81	.419
Shanxi	3.21 (−0.41 to 6.97)		1.73	.082
Inner Mongolia	−2.26 (−3.92 to −0.56)		−2.60	.009
Northeast region	−4.09 (−5.60 to −2.56)		−5.17	<.001
Heilongjiang	−5.82 (−8.09 to −3.48)		−4.80	<.001
Jilin	−5.21 (−6.29 to −4.12)		−9.17	<.001
Liaoning	−1.06 (−3.40 to 1.33)		−0.87	.382
Northwest region	−5.64 (−7.48 to −3.75)		−5.75	<.001
Shaanxi	−9.23 (−10.96 to −7.48)		−9.91	<.001
Gansu	−8.31 (−12.63 to −3.77)		−3.52	<.001
Qinghai	−0.45 (−2.73 to 1.88)		−0.38	.701
Ningxia	−9.84 (−11.37 to −8.28)		−12.89	<.001
Xinjiang	0.36 (−3.43 to 4.30)		0.18	.854
Eastern region	0.49 (−0.82 to 1.81)		0.73	.466
Shanghai	3.81 (1.12 to 6.56)		3.04	.008
Jiangsu	−3.61 (−5.11 to −2.06)		4.57	<.001
Zhejiang	−8.43 (−11.25 to −5.52)		5.51	<.001
Fujian	0.18 (−1.0 to 1.41)		0.29	.771
Shandong	3.19 (−0.45 to 6.96)		1.71	.087
Anhui	5.46 (4.35 to 6.59)		9.81	<.001
Jiangxi	0.93 (−0.97 to 2.87)		0.96	.339
Central region	0.84 (−3.79 to 5.69)		0.35	.727
Henan	−3.00 (−8.32 to 2.64)		−1.06	.291
Hubei	-0.38 (−2.69 to 1.98)		−0.32	.750
Hunan	10.52 (7.91 to 13.20)		8.19	<.001
Southern region	4.07 (1.41 to 6.80)		3.02	.002
Guangdong	4.40 (2.01 to 6.83)		3.66	<.001
Hainan	1.38 (−2.62 to 5.55)		0.67	.505
Guangxi	3.22 (0.83 to 5.67)		2.65	.008
Southwest region	3.60 (−4.86 to −2.33)		−5.48	<.001
Chongqing	−9.23 (−11.15 to −7.28)		−8.9	<.001
Sichuan	−4.47 (−6.47 to −2.43)		−4.24	<.001
Guizhou	0.30 (−3.41 to 4.14)		0.15	.877
Yunnan	−2.52 (−5.40 to 0.45)		−1.67	.094
Xizang	16.93 (14.31 to 19.60)		14.73	<.001
Whole country	−0.15 (−1.77 to 1.49)		−0.18	.855

a AAPC: average annual percentage change.

### Age-Period-Cohort Model

#### Age Effect

**Figure 3.**
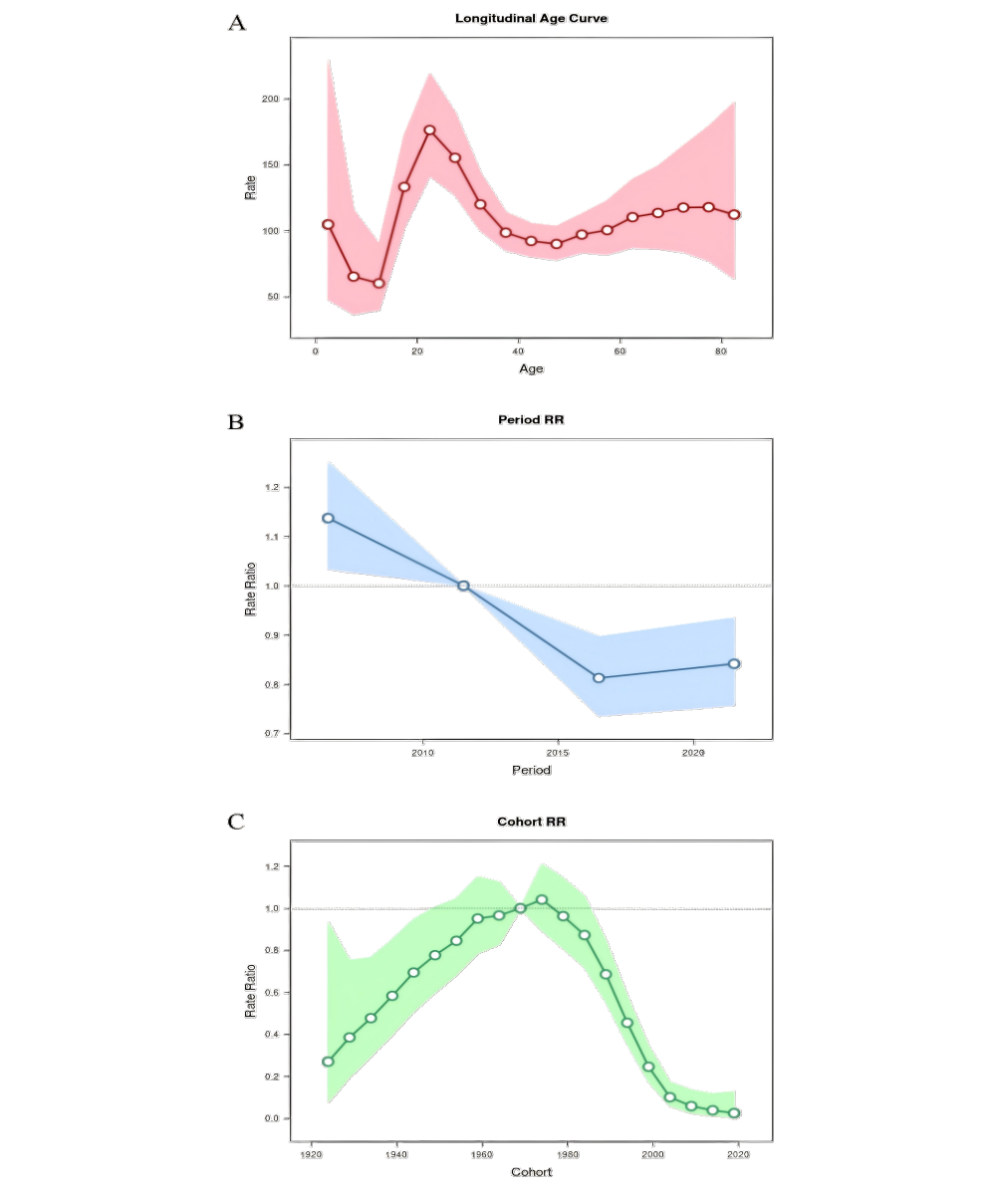
Average annual percentage change model of hepatitis B incidence rate in China from 2004 to 2020: (A) longitudinal age curve; (B) period rate ratio; (C) cohort rate ratio. RR: relative risk.

APC model analysis indicates that the majority of hepatitis B cases occur in adults, with the incidence rate exhibiting fluctuations across different age groups. The incidence rate increased from childhood to adolescence. At the age of 22 years, the incidence rate of hepatitis B reached its peak, with an average incidence rate of 176.173/100,000. Subsequently, it began to decline, remained relatively stable in middle age, and slightly increased in old age (Figure 3A).

#### Period Effect

After adjusting for age and cohort effects, the relative risk (RR) value for the period effect of hepatitis B incidence rate from 2004 to 2020 exhibited a trend of initially decreasing and then increasing. Using 2011 as the reference period (RR=1), the peak incidence rate of hepatitis B was observed between 2004 and 2006, with an RR value of 1.14 (95% CI 1.03-1.25). Subsequently, the RR value for the period effect on the incidence rate of hepatitis B exhibited a decreasing trend, with the RR value for the incidence rate of hepatitis B during the period from 2011 to 2016 being 0.81 (95% CI 0.74-0.9). The RR value of hepatitis B incidence from 2016 to 2020 was 0.84 (95% CI 0.76-0.93), indicating a slight increase compared with the previous period (Figure 3B).

#### Cohort Effect

After adjusting for age and period effects, the cohort born in 1969 was set as the reference cohort (RR=1). From 1924 to 1974, the RR value of the cohort effect for hepatitis B in China exhibited an increasing trend, reaching its peak in 1974 with an RR value of 1.04 (95% CI 0.9-1.21). The RR value of the cohort effect for hepatitis B in China decreased after 1974, reaching its nadir in the 2019 cohort, with an RR value of 0.03 (95% CI 0.01-0.13; Figure 3C).

### Spatial Distribution Characteristics of Hepatitis B in China From 2004 to 2020

**Figure 4.**
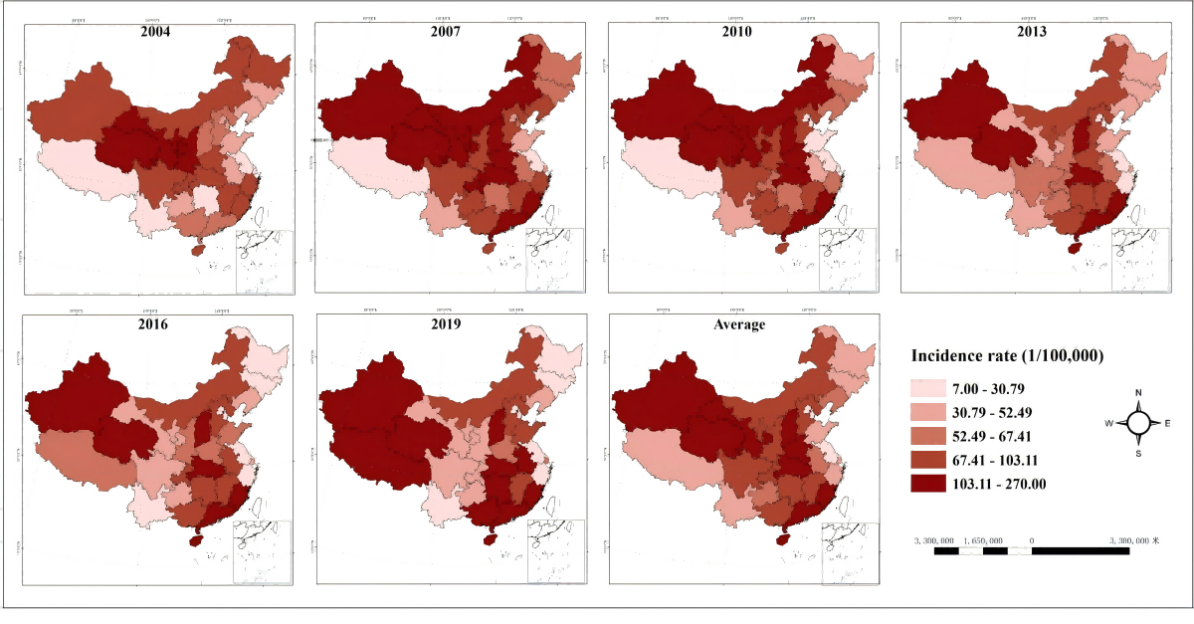
Spatial distribution characteristics of hepatitis B in China from 2004 to 2020.

Between 2004 and 2020, China witnessed significant regional disparities in the incidence rate of hepatitis B. The high-incidence regions of hepatitis B are primarily located in the northwest and central regions, while the incidence in the southwest and some parts of the eastern regions is gradually increasing over time. Among them, Qinghai (216.86/100,000), Xinjiang (170.21/100,000), and Gansu (130.23/100,000) exhibited the highest average incidence rate, whereas Jiangsu (18.56/100,000), Beijing (20.68/100,000), and Tianjin (23.54/100,000) had lower average incidence rate (Figure 4).

### Spatial Autocorrelation Analysis of Hepatitis B in China From 2004 to 2020

The global spatial autocorrelation analysis of hepatitis B incidence in 31 regions of China spanning from 2004 to 2020 revealed that the overall Moran’s *I* values for the periods of 2004 to 2011 and 2020 were greater than 0, ranging from 0.1089 to 0.2084 (all *P*<.05), indicating a positive spatial autocorrelation. No significant spatial autocorrelation was observed from 2012 to 2019 (all *P*>.05; Table 2).

**Table 2.** Global spatial autocorrelation analysis of hepatitis B in China from 2004 to 2020.

Year	Moran’s *I*	Expected index	Variance	Z values	*P* value
2004	0.2084	−0.0333	0.0057	3.2149	.001
2005	0.2024	−0.0333	0.0056	3.1603	.002
2006	0.1499	−0.0333	0.0056	2.4583	.014
2007	0.1304	−0.0333	0.0055	2.2073	.027
2008	0.1319	−0.0333	0.0053	2.2678	.023
2009	0.1089	−0.0333	0.0047	2.0844	.037
2010	0.1158	−0.0333	0.0054	2.0309	.042
2011	0.1227	−0.0333	0.0056	2.0895	.037
2012	0.0816	−0.0333	0.0055	1.5496	.121
2013	0.0581	−0.0333	0.0054	1.2471	.212
2014	0.1019	−0.0333	0.0057	1.7838	.074
2015	0.1109	−0.0333	0.0058	1.8991	.058
2016	0.0759	−0.0333	0.0057	1.4872	.149
2017	0.1082	−0.0333	0.0057	1.8735	.061
2018	0.1009	−0.0333	0.0058	1.7582	.079
2019	0.0999	−0.0333	0.0058	1.7432	.081
2020	0.1308	−0.0333	0.0058	2.1482	.032

**Figure 5.**
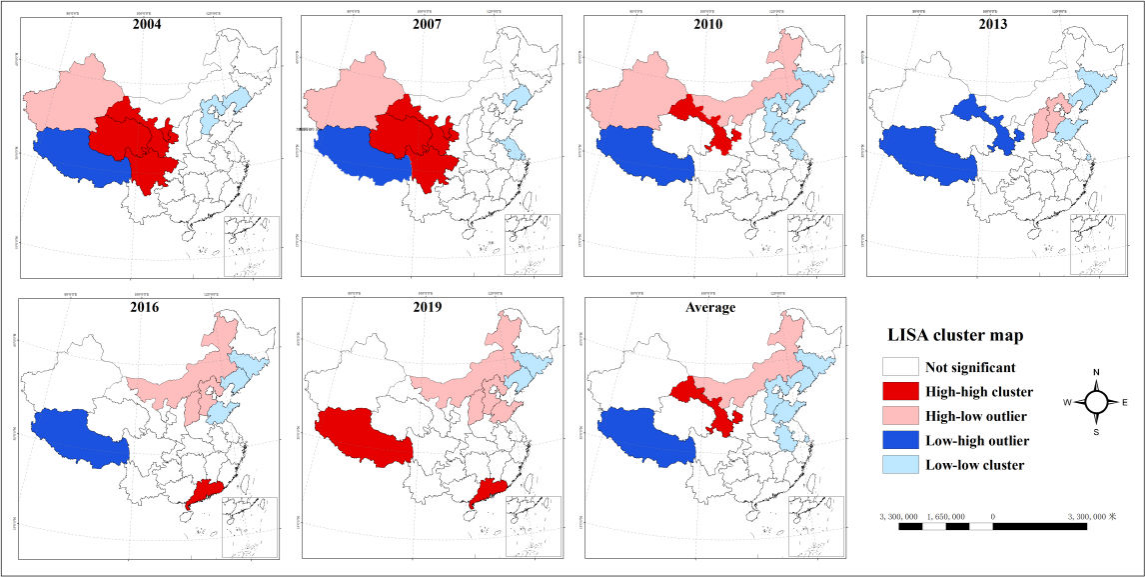
Local spatial autocorrelation analysis of hepatitis B in China from 2004 to 2020.

The results of local spatial autocorrelation analysis indicate that low-low cluster regions are primarily located in Jilin and Liaoning in Northeast China, as well as Shandong in the eastern region. The high-low cluster regions have shifted from Xinjiang in the northwest to northern regions such as Inner Mongolia and Hebei. From 2004 to 2007, high-high cluster regions were primarily located in the northwest and southwest regions, specifically in Qinghai, Gansu, Ningxia, Sichuan, and Chongqing. After 2007, the high-high cluster regions phenomenon gradually dissipated in the northwestern region of China, except Ningxia. In 2013, Ningxia transformed from a high-high cluster region to a high-low cluster region, ultimately evolving into a region with no significant spatial clustering. Since 2015, Guangdong Province has transformed from a region with no significant spatial clustering to a high-high cluster region, whereas Xizang has shifted from a high-low cluster region to a high-high cluster region (Figure 5).

## Discussion

### Principal Findings

Using joinpoint regression analysis, the APC model, and spatial autocorrelation analysis, this study delves into the epidemic characteristics and spatial distribution patterns of hepatitis B in China from 2004 to 2020. The findings offer valuable data support and theoretical insights for comprehending the evolving epidemic trends of hepatitis B among Chinese residents and formulating targeted prevention and control strategies accordingly.

The study findings revealed that the overall incidence rate of hepatitis B in China exhibited a decreasing trend between 2004 and 2020, with a decrease from 70.5/100,000 in 2004 to 64.29/100,000 in 2020. This indicated that the prevention and control measures for hepatitis B in China from 2004 to 2020 had yielded certain positive outcomes, which align with the findings of other studies [17]. According to the results of joinpoint regression analysis, the incidence rate of hepatitis B in China exhibited an increasing trend between 2004 and 2007, subsequently experienced a decreasing trend, and ultimately stabilized after 2014. Since 2007, the incidence rate of hepatitis B decreased, primarily attributed to the neonatal hepatitis B vaccination program that was launched in China in 1992 [18,19]. Furthermore, China has been offering free hepatitis B vaccinations to newborns since 2002, significantly reducing the risk of hepatitis B infection among newborns. A study revealed that the hepatitis B vaccination coverage among individuals aged 1 to 59 in China increased significantly, escalating from 54.1% to 78.9% between 2006 and 2024 [20]. According to a study on the global burden of disease, the positive rate of HBsAg in China has undergone a significant decrease, decreasing from 11.9% in 1990 to 8.4% in 2015, and further decreasing to 7.8% in 2019 [7]. These data provide further evidence of China’s significant advancements in the prevention and control of hepatitis B.

The APC model-based analysis of age effects revealed that the risk of hepatitis B is relatively low in childhood, rises during adolescence, peaks at age 22, and exhibits a decreasing trend in older adulthood. The increased incidence rate among young adults may be attributed to high-risk sexual behaviors (such as unprotected sex and multiple sexual partners), unhealthy lifestyles (including excessive stress, staying up late, alcohol abuse, and smoking), and specific occupational exposures [21,22]. The increased risk of hepatitis B in the elderly is significantly influenced by the low coverage of hepatitis B vaccination in this population. A study exploring the factors influencing hepatitis B vaccination coverage highlighted a negative correlation between vaccination coverage and age, which is likely linked to the national hepatitis B immunization program for newborns and infants instituted by the Chinese government in 1992 [23]. In addition, the decrease in immune function among the elderly further heightens their susceptibility to hepatitis B infection [24]. In terms of period effects, the highest hepatitis B incidence rate in China during 2004‐2020 was observed between 2004 and 2006. Cohort analysis indicated that individuals born between 1924 and 1974 exhibited an increased trend in hepatitis B incidence risk, whereas a decreased trend was observed among those born after 1974. These shifts in period and cohort effects can be attributed to factors such as the widespread adoption of hepatitis B vaccines, advancements in medical technology, increased public awareness of hepatitis B prevention, and changes in lifestyle habits [25]. Therefore, an in-depth discussion of the age, period, and birth cohort effect of hepatitis B can not only help to locate the high-risk population of hepatitis B, but also provide important references for the formulation of public health policies in the future, to more effectively control the spread and risk of hepatitis B.

The overall incidence rate of hepatitis B in China exhibited a decreasing trend from 2004 to 2020, albeit with regional variations in its trajectory. Specifically, the northeastern and northwestern regions observed a decrease in hepatitis B incidence. Notably, the northwestern region experienced a steeper decrease in hepatitis B incidence rate compared with the northeastern region. However, the northwestern region consistently maintained a relatively high incidence rate, particularly in Qinghai, Xinjiang, and Gansu, where the average incidence rate from 2004 to 2020 ranked among the highest nationwide. This high incidence rate can be attributed to various factors such as geographical conditions, sluggish economic development, scarcity of medical resources, and varying levels of health literacy among residents [12]. Contrastingly, the southern and southwestern regions witnessed an increased trend in hepatitis B incidence. This increasing trend in the southern region is likely associated with factors like frequent population movements, uneven distribution of health care resources, and geographical considerations. Given the dense and mobile population in the southern region, disparities in health literacy, access to health care resources, and vaccination coverage among the mobile population may contribute to the widespread transmission of the HBV in this region. In addition, the humid and hot climate prevalent in the southern region may facilitate the easier spread of hepatitis B [26]. In addition, Liang et al [27] showed that some alleles are closely related to HBV infection. There are obvious differences in genotype distribution between southern and Northern Chinese populations. The difference in genetic background may be a potential factor for Southern Chinese populations to be more susceptible to hepatitis B. Simultaneously, the increasing incidence rate in Southern China in recent years can also be partially attributed to the enhanced quality of hepatitis B case reporting, which has significantly reduced previously overlooked cases [12,28]. The swift increase trend observed in Southwest China is primarily attributed to the rapid incidence rate in Xizang. Located on the southwestern border of China, the Xizang, owing to its vast territory, sparse population distribution, and inconvenient transportation, lags behind other provinces in immunization planning. Consequently, Xizang has consistently been identified as a high-prevalence area for hepatitis B [29].

Through global spatial autocorrelation analysis, this study revealed the spatial distribution characteristics and evolving trends of hepatitis B incidence in 31 regions of China from 2004 to 2020. The findings of the global spatial autocorrelation analysis indicated that there was a significant clustering of hepatitis B incidence rate in China during the periods of 2004‐2011 and 2020. These cluster regions could potentially be attributed to socioeconomic disparities, the distribution of medical resources, varying health conditions, and the implementation of preventive measures across different regions [30]. Based on the data from 2012 to 2019, no significant spatial autocorrelation was evident, indicating a more randomized geographical distribution of hepatitis B during this period and a narrowing of incidence rate disparities among regions. These changes may be attributed to the full implementation of “China’s viral hepatitis prevention and control plan (2012‐2020)” for gradually reducing the differences in hepatitis B prevalence between regions, as indicated by the high overall hepatitis B vaccination rate for newborns, which has been maintained at >90%; the popularization of hepatitis B prevention and control knowledge among the population; and the effective construction of the population immune barrier.[31].

Local spatial autocorrelation analysis has further illuminated the clustering patterns of hepatitis B incidence rates across diverse regions. Specifically, certain regions in the eastern part of the northeast exhibit low-low cluster regions, whereas regions in the northwest demonstrate high-low cluster regions. According to the findings of Ren et al [32] on the allocation of medical and health resources in China’s provinces from 2010 to 2021, there has been an increasing trend in medical and health resources, and the allocation has essentially achieved parity in terms of quantity. However, high-quality medical and health resources in China are heavily concentrated in the eastern region, which may further explain the disparities in the spatial pattern of hepatitis B incidence among regions. Between 2004 and 2007, northwest and southwest regions, including Qinghai, Gansu, Ningxia, Sichuan, and Chongqing, exhibited high-high cluster regions. Nevertheless, this spatial aggregation gradually dissipated in 2007. This shift could be attributed to significant progress made in hepatitis B prevention and control efforts in these regions, leading to changes in the spatial clustering pattern of the disease. Since 2015, Guangdong and Xizang have transformed from areas with nonsignificant spatial cluster regions to high-high cluster regions. This transformation is likely linked to population movements, economic development, and improvements in health conditions in these regions. As an economically developed region, Guangdong Province has frequent population mobility, which is an important reason for increasing the risk of hepatitis B transmission [33]. However, due to the improvement of health conditions, the increase of medical resources, and the improvement of diagnostic levels in Xizang, the detection and reporting of hepatitis B is more accurate, thus showing a high-high clustering phenomenon [29].

This study offers fresh perspectives on the epidemic trends and spatial clustering patterns of hepatitis B in China, serving as a scientific foundation for developing targeted prevention and control measures. Future research should delve deeper into the factors influencing the variations in hepatitis B incidence rates across different regions, as well as explore more effective strategies for implementing preventive measures, particularly in regions with high incidence rates or evident increase trends. Concurrently, it is imperative to enhance vaccination and health education efforts targeting adults to mitigate the occurrence of hepatitis B.

This study has several limitations in analyzing hepatitis B surveillance data in China, which are worthy of further discussion. First of all, this study was limited to the analysis of the incidence of hepatitis B in China for 17 years (2004 to 2020) and failed to explore the influencing factors of the incidence of hepatitis B. In the future, a longitudinal research design should be considered to systematically assess the impact of various potential factors on the incidence rate of hepatitis B. Second, due to the limitations of data and methods, this study failed to fully analyze the differences between genders, differences between occupations, differences between urban and rural areas, and other factors that influence the incidence rate of hepatitis B in China. Therefore, future research should further improve the data and conduct a hierarchical analysis of these factors, to identify high-risk groups and develop targeted interventions. In addition, although economic factors, population density, medical service level, and medical resource allocation have an impact on the spatial clustering of hepatitis B, the impact of these factors on the regional incidence level still needs further research.

### Conclusions

From 2004 to 2020, China witnessed a decrease in the overall incidence rate of hepatitis B, indicating remarkable progress in its hepatitis B prevention and control efforts. Nevertheless, challenges persist in specific populations and regions. Therefore, it is imperative to maintain vigilance in hepatitis B prevention and treatment, in addition to enhancing health education initiatives. Further, given the disparities in age and geographical distribution, efforts must be intensified to identify and screen high-risk groups and promote supplementary vaccination programs for adults. These comprehensive measures will alleviate the health burden of hepatitis B on the population, enhance the overall standard of healthy living, and establish a robust scientific foundation for the ongoing prevention and control of hepatitis B in China.

## Abbreviations

AAPC: average annual percentage change
APC model: age-period-cohort-model
HBsAg: hepatitis B surface antigen
HBV: hepatitis B virus
HCC: hepatocellular carcinoma
RR: relative risk
